# Highlights from the 2014 International Symposium on HIV & Emerging Infectious Diseases (ISHEID): from cART management to the end of the HIV pandemic

**DOI:** 10.1186/1742-6405-11-28

**Published:** 2014-08-21

**Authors:** Alain Lafeuillade, Mark Wainberg, Marie-Lise Gougeon, Sabine Kinloch-de Loes, Philippe Halfon, Hervé Tissot-Dupont

**Affiliations:** 1 Department of Infectious Diseases, General Hospital, Toulon, France; 2 McGill University, Montreal, Canada; 3 Antiviral Immunity, Biotherapy and Vaccine Unit, Infection and Epidemiology Department, Pasteur Institute, Paris, France; 4 Royal Free Center for HIV Medicine, Department of Infection and Immunity, Royal Free Hospital, London, UK; 5 Internal Medicine and Infectious Diseases Department, European Hospital and Alphabio Laboratory, Marseille, France; 6 Health Department, Research Institute for Development, Marseille, France

**Keywords:** HIV, HCV, Hepatitis E, Antiretroviral drugs, HIV vaccine, HIV cure, HIV reservoirs

## Abstract

The 2014 International Symposium on HIV and Emerging Infectious Diseases (ISHEID) provided a forum for investigators to hear the latest research developments in the clinical management of HIV and HCV infections as well as HIV cure research. Combined anti-retroviral therapy (c-ART) has had a profound impact on the disease prognosis and transformed this infection into a chronic disease. However, HIV is able to persist within the infected host and the pandemic is still growing. The main 2014 ISHEID theme was, hence “Together for a world without HIV and AIDS”. In this report we not only give details on this main topic but also summarize what has been discussed in the areas of HCV coinfection and present a short summary on currently emerging viral diseases.

## Introduction

The 2014 International Symposium on HIV and Emerging Infectious Diseases (ISHEID) was held in Marseille, France, on May 21-23. This meeting attracted more than 900 delegates from all over the world (75% outside France) and allowed interactive discussions around HIV, viral hepatitis, new influenza strains, MERS-CoV and Chikungunya infection. In this article we focus on some aspects of these discussions.

## Antiretroviral therapy

From the point of view of HIV infection, the emphasis was very much on antiretroviral therapy with new compounds in development and results of recent Phase III trials as well as treatment issues in the young such as adolescents and less young in an aging epidemic in what has become a chronic disease. How to end the epidemic, the development of a prophylactic vaccine and HIV eradication took the front stage on several occasions during these three days.

The conference started by “The Thirty Years of Discoveries of HIV” and “The Ten Big Mistakes We Did”. Stefano Vella reminded us of the hopes in the early days of the epidemic of a prophylactic vaccine within the next 3 years. This prediction probably refers on Margaret Heckler declaration in 1984 [1] but what she said is that as we were able to grow HIV, this would help finding a vaccine one day. Vella also listed the short-life of the experiment in treatment interruptions and, to the contrary, the prolonged use of D4T, and the delay in using surrogate markers and understanding the ART-associated side-effects such as lipodystrophy. We also took time to take into account the impact of ART as a preventative measure while the exact timing of ART initiation is still being assessed with in ongoing studies. Stefano Vella concluded that the understanding of HIV pathogenesis and the development of new drugs paved the way for the dramatic progress of the 1990s in terms of morbidity and mortality with combination therapy. Nearly 10 million of individuals are now on treatment worldwide and the appalling discrepancy in access between rich and developing countries is progressively being filled. This process is also exemplified by the 2013 WHO Guidelines for the Treatment of Adults which have aligned themselves with those from other international committees.

Mark Wainberg [2] spoke about future challenges with a strong emphasis on finding a cure for HIV infection. Current efforts to provide ARVs to the millions of people infected by HIV in developing countries may not be sustainable. Professor Wainberg hypothesized that dolutegravir is a qualitatively superior drug than any of its predecessors and that drug resistance against dolutegravir may be impossible if this compound is used in first line therapy for reasons of efficient drug binding to the integrase enzyme and greatly reduced viral fitness if viruses do become minimally resistant. He stated that patients on dolutegravir may be unable to transmit HIV and that the use of this drug may therefore contribute to ending the HIV epidemic.

The sessions on the management of antiretroviral therapy reminded us that there is a robust pipeline of new compounds in development. Roy Gulick [3] discussed the need of new NRTIs with activity against resistant viral strains and less long-term toxicity. Tenofovir alafenamide fumarate known as TAF is under development and shows a viremia decrease of about 1 log over 10 days dosing and should display a safer toxicity profile in terms of kidney and bone than tenofovir. Doravirine, a potent new NNRTI which is metabolized by CYP3A4 and active *in vitro* against viral strains with K103N, Y181C, G190A, E101K, E138K or K103N/Y181C is in development. The attachment inhibitor class has an interesting one daily compound active on both CCR5 and CCR2, cenicriviroc, which has also potential anti-inflammatory properties. BMS-663068 is an attachment inhibitor with a new mechanism of action. It binds to the envelope protein gp120 and is therefore different form the CCR5 class of drugs. It is active against most HIV strains but not all, is potent and well tolerated.

The important results from The LATTE [3] study which showed that rilpivirne and GSK 1265744 (744) orally in maintenance therapy suppressed viremia are paving the way for the testing of injectable forms of this combination which might be deliverable on a monthly basis. Its use as a pre-exposure prophylaxis agent will also be tested soon. The exciting development of injectable long-acting ARV’s was presented by Marta Boffito [4].

In terms of first line therapy in 2014 Marc Nelson [5] presented the positive results from the recent trials of newly developed antiretroviral drugs such as those of phase III of elvitegravir and dolutegravir and stressed the favourable resistance profile of dolutegravir together reminding us of the mechanism of action at the tubular level of the booster cobicistat. Delays had occurred in detecting many ART-associated toxicities. Drug costs are at the forefront of the treatment agenda and generic formulations for many antiretrovirals are available or on the way which will have important impact on drug cost in the future. A trial of first line therapy with lopinavir/ritonavir (LPV/r) vs nevirapine and 2 NNRTIs in a developing country: W144 of a randomized trial by Nathan Clumeck [6] showed favourable outcome for the protein inhibitor-based regimen.

In the discussion on switch strategies Daniel Podzamer [7] reviewed the causes for switching ART within the context of imperfect ART with adherence problems, toxicity among others. He reminded us that not all switch strategies have been tested.

## HIV as a chronic disease

Some of the important issues that clinicians encounter nowadays with HIV as a chronic disease are characterized by processes which are associated with increased morbidity and morbidity and are not related to the traditional AIDS-defining events, such as cardio-vascular, renal, metabolic and bone diseases. Chronic inflammation as discussed by Frank Miedema [8] is one of the critical factors in HIV pathogenesis and the most likely cause of non-AIDS end-organ disease encountered during chronic HIV infection such as atherosclerosis, cardio-vascular disease, kidney failure and neuropathology. This process remains active even during combination ART. In Aging with HIV Paddy Mallon [9] reviewed the various markers of immune dysfunction and changes associated with ageing and HIV infection. The ageing process and HIV infection are associated with similar changes beside immune dysfunction such as bone changes and cardio-vascular disease. The rational conclusion regarding co-morbidities in HIV-patients was to address them early and to have a long view regarding the choice of ART regimen taking into consideration all these factors according to Barry Peters [10]. The place of statins was reviewed by Kenneth Lichtenstein [11]. His conclusion was that in terms of risk of type 2 diabetes their benefit outweighed the risk. Lipids levels also are not the whole story and stressed the importance of lifestyle changes such as weight reduction and smoking cessation.

On the other side of the age spectrum we are dealing with a young population either infected vertically or horizontally due to improved treatments children born with HIV reach adulthood. Rana Chakraborthy [12] reiterated the importance of organizing the transitioning process of HIV Infected Youths into the Adult Healthcare system using multidisciplinary individualized approaches and the different needs regarding in particular stigma, mental health and high rates of teen pregnancy that these two populations have in terms of support.

## Ending the HIV pandemic

Last but not least, how to stop the epidemic and achieve a cure? Comprehensive reviews were provided by Virginie Supervie and Francois Dabis [13] as well as Myron Cohen [14]. We need to track the current state of the epidemic, early diagnosis of the HIV infection, prevent the acquisition and the transmission of HIV. Our preventative tools have grown in the past few years with the acknowledgment of the impact of ART as a tool for prevention. Myron Cohen discussed the different prevention strategies and interventions in the uninfected and infected individuals. Factors associated with amplified transmission include those associated with infectiousness such as blood and genital tract viral load, sexually transmitted diseases (STDs), viral clade and acute infection and susceptibility for the acquisition of the virus such as STDs among others. Joep Lange [15] reiterated the strong arguments in favor of early therapy and its individual and public health benefits in the implementation of TasP. Francois Dabis’ recommendations for ending the epidemic within the next 20 years were to go to the communities, counsel and test, treat everybody, promote condom use and male circumcision and harm reduction.

Is there a possibility of a cure for HIV? In his update on reservoirs Lian Shan [16] showed the complexity of HIV-1 integration, in particular in memory precursor CD4^+^ T cells and the potential role of CTLs boosting in the elimination of infected cells. The pharmacological approaches by Ole Schmeltz Sogaard [17] covered multiple immmunologcial interventions including neutralising antibodies beside therapeutic HIV vaccines, Anti-PD-L1 Ab, NK cells, peg INF-alpha and TLR agonists and others. Gene therapy and bone marrow transplantation were discussed by Joachim Hauber and Jan van Lutzen [18]. Gene therapy has had limitations because of the low number of transduced cells and genotoxicity. Zinc finger nucleases (ZFN) technology has the disadvantages of the possibility of off-target effect and chromosome instability. Joachim Hauber [19] presented the next generation LTR-specific Tre-recombinase which targets a majority of HIV-1 isolates. It attacks the reservoir directly and is presently expanding to other subtypes. Anne Gatignol [20] discussed the new therapeutic target in Gag RNA accessible to ribozyme and RNA interference molecules and in particular hepatitis delta-derived ribozyme to target HIV-1 RNA with long-term inhibitory activity suggesting that this type of molecules might be used in combination gene therapy or as drugs.

The development of an HIV/ AIDS vaccine has been a long and difficult endeavor, which so far was met with many failures. The hopes and challenges in HIV vaccine research were discussed at the 2014 ISHEID Conference. Vaccine challenges include the genetic diversity and mutability of HIV-1 that create a plethora of constantly changing antigens, the structural features of the viral envelope glycoprotein that disguise conserved receptor-binding sites from the immune system, and the presence of carbohydrate moieties that shield potential epitopes from antibodies. Indeed, HIV-1 gp120 contains a number of features that help the virus evade the host’s humoral immunity, including variable loops, N-linked glycosylation, and conformational flexibility that disguise the conserved receptor-binding sites from the humoral immune system [2]. The presence of carbohydrate moieties on gp120 shields potential epitopes from binding to antibodies, as shown by Czernekova et al. [21]. By partially removing N-glycans from gp120 they increased the reactivity of a panel of gp120-specific broadly neutralizing antibodies and sera from HIV-1-infected persons.

The successful development of an HIV vaccine is one of the ten big challenges highlighted by Marc Wainberg [2]. He also pointed out that there is a very limited rate of success in the development of vaccines to all forms of sexually transmitted disease, with the obvious exception being Human Papilloma Virus [2]. As reviewed by Marc Girard [22], the first efficacy trials for an HIV vaccine attempting to elicit protective antibodies against the viral envelope, VAX004 and VAX003, did not prevent HIV-1 infection or impact the level of viraemia in those infected. The next set of antigens used in clinical efficacy studies were designed to test whether T cells could protect against HIV-1 infection. Neither the STEP trial (Ad5 *gag/pol/nef*) nor HVTN 505 trial (DNA/rAd5 *gag*/pol*/nef/env* prime/boost regimen) could protect against HIV-1 infection, or reduce early plasma HIV-1 levels. The proof of concept that it is possible for a vaccine to elicit protective immunity that blocks infection came from the RV144 clinical trial in Thailand that yielded a 31% reduction in HIV-1 acquisition using a canarypox-gp120 prime-boost vaccination regimen [23]. Moreover, as highlighted by Marc Girard, new surrogate markers of protection came from the RV144 trial, including the presence of V1-V2 targeted IgG. The V2 loop contains the binding site to the α4β7 receptor of CD4+ T cells involved in T cell migration to the GALT, and it is also part of several neutralization epitopes, including those recognized by broad neutralizing antibody (bNAb) VRC26.08 at the apex of the HIV-1 spike [24]. The RV144 trial also showed strong correlation between protection and IgG that target the C1 domain of gp120 and promote ADCC [25]. Several studies that addressed the question of correlates of protection from infection or from the disease were reported at the ISHEID Conference. The resistance to HIV-1 infection among exposed seronegative partners in HIV-discordant couples was associated with higher frequency of CD8^+^ T cells expressing CD107a/b and IFN- ascompared to unexposed subject [26], suggesting a protective role of HIV-specific CTLs. The protective role of CTLs was also suggested by the finding that HIV controllers exhibit high frequency of CD8^+^ T-cells displaying the phenotype CD38^neg^HLA-DR^+^ and exerting *ex-vivo* cytotoxic activity, the frequency of these cells being correlated with the capacity of CD8^+^ T cells to inhibit viral replication [27]. A novel experimental approach was presented to analyze lymphocytes from post treatment controllers (PTC), in particular the presence of replication competent HIV, following transplantation of Rag2^-/-^c^-/-^ (Rag-hu) mice with CD4^+^T cells from the PTC and subsequent engraftment with human T cells [28].

Broadly reactive neutralizing antibodies (bNAbs) have been found in select individuals living with HIV, demonstrating that humans are capable of making broadly neutralizing antibodies [29]. They show potent cross-clade neutralizing activity against 70-90% of HIV-1 strains. They are remarkably protective against i.v. or mucosal HIV or SHIV infections in animal models, and they can suppress active infections in mice and macaques. bNAbs develop over a period of a few years (2.5 years average) in only 15-20% of HIV-1 infected persons, and they are the result of a long affinity maturation of B cells and extensive mutation of the B cell lineage that seem to be driven by long antigenic exposure [30]. A vaccine should offer protection against the majority of HIV strains. Hence, a number of questions should be addressed: i) are we able to design a vaccine that induces rapid production of V1V2-specific protective Abs? ii) how to transfer the knowledge of cross-specific antigens into immunogens that will induce powerful protective response following vaccination? iii) do we know enough about B cell maturation and plasmocyte differentiation? iv) which immunogen to develop considering that broadly neutralizing Abs that were identified are hypermutated ? it may be needed to stimulate a succession of antibodies that recognize epitopes and neutralize them in a series of vaccination; v) how to induce the correct B cells and stimulate them to produce Abs with a high degree of affinity maturation ? vi) how to induce humoral memory that is mediated by long-lived antibody-secreting cells? vii) successful vaccines (smallpox, hepatitis A, YF) elicit broad integrated responses involving all effectors, including innate (TLRs), NK, Th1/Th2 responses. Considering that innate immunity influences both the qualitative and quantitative features of adaptive immunity, we should identify the innate correlates of protection.

The question still arises whether a vaccine that does not prevent infection but reduces HIV levels and preserves uninfected memory CD4^+^ T cells would benefit the recipient. Several studies were presented at ISHEID conference reporting the development of new therapeutic vaccine candidates and their safety in preclinical and clinical trials. A new technology was reported, conferring one-step *in vivo* production in targeted cells of an efficient and safe DNA-virus-like particle (VLP) expressing gagV3(BCE), and leading to the presentation of immunodominant HIV antigens [31]. In preclinical studies, GenePro, a lentiviral therapeutic vaccine that includes 7 out of 9 HIV genes, was able to induce in a single high dose a long-lasting and polyfunctional T-cell response in the nonhuman primate model [32]. Another lentiviral-based therapeutic vaccine was presented, generating a strong, specific and long-lasting T-cell response in mice, and currently evaluated in HAART-treated patients in a phase I/II vaccine trial [33]. A different approach consisted in the induction of neutralizing antibodies to HIV-1 matrix protein p17 that, through the interaction of its NH2-residing epitope AT20 with specific receptors, acts extracellularly as a viral toxin. A peptide-based immunogen, AT20-KLH, was used in a phase I trial and shown to generate p17-specific antibodies able to neutralize its binding to cell receptors in HAART-treated patients, and still detected two years after the first injection [34].

Finally, a novel approach to protect with antibodies against an infectious disease was recently developed: the vector-immunoprophylaxis (VIP) or Vector-mediated Ab gene transfer, which aims at eliciting passive immunization using an AAV vector that expresses the H and L chains of a monoclonal bNAb (such as VRC-01, VRC-07…). This ‘gene-therapy’ approach has been successfully tested in animal models (BLT mice) conferring a full protection against mucosal HIV transmission [35], suggesting that a sufficiently high circulating concentration of bNAb might substantially reduce the probability of sexual transmission of HIV between humans.

The search for a HIV vaccine is a long story, starting in the early 90s by the first clinical trial performed in humans by Daniel Zagury [36] and a long road is still ahead. Promizing strategies are, however, in the pipeline [37].

## Towards a HCV eradication

Hepatitis C virus (HCV) is a major cause of chronic liver disease, with an estimated 170 million people infected worldwide [38]. HCV, identified in 1989, is an enveloped virus with a 9.6 kb single-stranded RNA genome, a member of the Flaviviridae family, genus Hepacivirus [39]. The development of new molecules called direct acting antivirals (DAA) leads to a complete change of the picture with the cure of the disease and a hope for an eradication. Overall, the future is bright for patients chronically infected with HCV. The challenge of the HCV disease is different for people regarding the location of their HCV infection. A major challenge in the Western world will be to identify most or all patients with HCV associated liver disease that would benefit from therapy. So far, only a minority of HCV-infected individuals is being diagnosed and of those, only a portion receives therapy. In the developing world, challenges will differ: making HCV therapies economically affordable in such areas of the world will become a major challenge.

The HCV lifecycle begins with virion attachment to its specific receptor The HCV RNA genome serves as a template for viral replication and as a viral messenger RNA for viral production. It is translated into a polyprotein that is cleaved by proteases. Then, viral assembly occurs. Potentially, each step of the viral cycle is a target for drug development. The knowledge of the structures of HCV protease and HCV polymerase has allowed structure-based drug design to develop inhibitors to these enzymes [40,41]. Several findings suggest that HCV modulation of IFN induction and signaling attenuates the expression of IFN stimulated genes, allowing HCV to escape the antiviral actions of the host response. All the HCV enzymes – NS2-3 and NS3-4A proteases, NS3 helicase and NS5B RdRp – are essential for HCV replication, and are potential drug discovery targets. Therefore DAA with different viral targets, including NS3 protease inhibitors, nucleoside/nucleotide analogue and non-nucleoside inhibitors of the RNA-dependent RNA polymerase, and NS5A inhibitors are under development.

Jügen Rockstroh described the progress made against HCV disease, with emphasis on drugs that combat HCV, which can now theoretically be considered to be curable since HCV does not integrate into cells. However, Professor Rockstroh [42] highlighted that HCV therapy has not yet been employed to sufficient extent among individuals who are not enrolled in controlled clinical trials. Clearly, it is possible that issues of non-adherence may compromise the success of anti-HCV drugs and that this, in turn, could lead to HCV drug resistance. Further work is necessary on this topic.

A lecture on new anti HCV agents by Fabien Zoulim [43] made clear that pharmaceutical companies are still active in this area. Professor Vicente Soriano [44] spoke about treatment of co-infected HCV/HIV patients and expressed hope that Sofosbuvir (SOF) would represent a huge improvement in our ability to treat HCV. Professor Marc Bourliere [45] spoke about new anti-HCV drugs would limit the use of the much more toxic Interferon/(IFN)/Ribivirin(RBV) approach that has dominated the field for decades.

**Figure 1:**
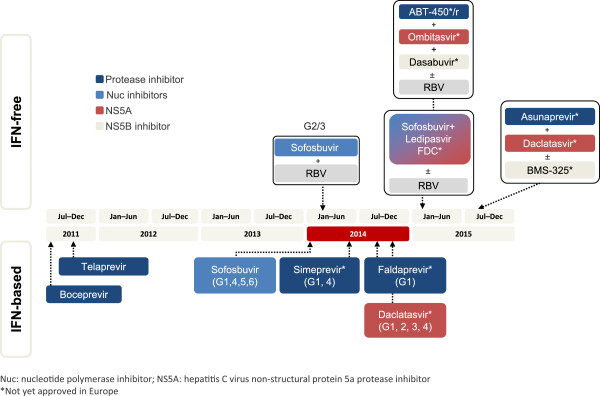
Improved HCV treatments continue to be developed.

There are key factors in deciding to treat a patient or to wait. Patients’ factors include urgency to treat, likelihood of response, HCV genotype, treatment experience, IL28B genotype, degree of fibrosis and patient motivation. Treatment factors include efficacy of current options, safety of current options, duration of therapy, pill burden, dosing frequency, future options and their timelines, access and cost.Simple as well as complex strategies are on development regarding the availability of the new DAA and the tolerance of IFN (Figure 1).

In genotype 1 patients, the combination of IFN + SOF has to be in balance with the IFN-free regimens based on Nucleoside Inhibitors (NI) + NS5A ± RBV or NI + Protease Inhibitor (PI) ± RBV. The combination of IFN + PI has also to be in balance with the IFN-free regimens such as PI + NS5A ± RBV or PI ± (Non Nucleoside Inhibitor) NNI + RBV or PI ± NNI + NS5A + RBV.

In genotype 2 patients the combination of SOF + RBV for 12 weeks of treatment leads to a 93-95% SVR and will be the next standard-of-care.

In genotype 3 patients the combination SOF + RBV for 24 weeks leads to a >90% SVR. Other DAA in combination are currently studied.

In genotype 4 patients the combination of SOF + RBV leads to a 79% response in naïve patients with 12 weeks of therapy and 100% with 14 weeks. The combination of the Abbvie drugs (ABT-450/r + ABT-267 ± RBV) leads to >90% SVR in both naïve and experienced patients.

In treatment experienced non-cirrhotic patients the combination of SOF + RBV for 24 weeks lead to 85% SVR. In cirrhotic patients, the combination of IFN + RBV + SOF for 12 weeks leads to 83% SVR.

## Emerging infectious diseases

According to Harry Dalton [46], hepatitis E must be considered as a common emerging disease in developed countries, where it is caused by genotype 3, mainly affects middle-ages/elderly males, and is zoonotic with a porcine primary host. However the route of transmission is not fully understood (no evident association with pigs in most of the cases). Blood transfusion must be considered accordingly to the high number of asymptomatic viraemic donors. It is now evident that HEV may cause persistent disease in immune-compromised solid organ transplant recipients, individuals with HIV and patients with haematological malignancy. Patients with chronic HEV infection have no symptoms, but some develop rapidly progressive liver cirrhosis. HEV has important extra-hepatic manifestations, which deserve further investigation. For example, Dalton et al have shown that HEV is neuropathogenic, and can cause a wide range of neurological illness.

As exposed by Bruno Lina [47], humans are usually not infected by avian influenza A viruses from aquatic wild birds. However, sporadic cases of avian influenza virus infection are observed every year, mostly in Asia. By subsequent adaptation of these emerging viruses may result into human pandemic strains.

Recently, a novel influenza A virus of the H7N9 subtype has been isolated from severely diseased patients with pneumonia and acute respiratory distress syndrome and, apparently, from healthy poultry in March 2013 in Eastern China. This virus is a result of a triple reassortment event that occurred in wild birds. Transmission to humans occurred in wet markets, mostly in large cities like Shanghai, in adults and elderly people, with a provisional fatality rate as high as 30% (122/413). No human to human transmission has been reported so far, which cannot be explained by the virological features of the H7N9 strains that harbours most of the known markers required to facilitate transmission in mammals. WHO experts consider this virus as an imminent threat.

Ten years after the SARS-CoV epidemic in Asia, a newly emerged Middle East lower respiratory tract syndrome (MERS-CoV) has been confirmed in 189 patients of which 82 had a fatal outcome. Marcel Müller [48] reports that all MERS cases were linked to the Arabian Peninsula and a few cases were imported into the European Union and Africa. Bats harbor a high diversity of related viruses and are suspected to be the animal reservoir. Recent studies have revealed that dromedary camels are the most likely intermediate host. MERS-CoV has been circulating in dromedary camels for decades raising the question whether earlier human cases may have remained undetected. Preliminary serological surveys in Saudi Arabia could not determine increased seroprevalence for MERS-CoV antibodies in the population. In-vitro studies showed that MERS-CoV inhibited important cellular pathways in particular the INF response. Confirmatory in-vivo experiments are still limited due to the lack of a suitable animal model, even non-human primates. A combination of pegylated IFN-alpha and RBV improved the clinical symptoms confirming previous in-vitro experiments. The development of vaccines is ongoing and vaccination programs in dromedary camels might be the optimal choice to control the current outbreak and prevent future re-introductions into the human population.

In 2006, during the major Chikungunya outbreak in La Reunion Island (Indian Ocean), many French military policemen were deployed. The French Military Health Service and Army Center for Epidemiology and Public Health could follow up those policemen 6 years after the outbreak [49]. They also studied the clinical spectrum and treatment of the rheumatic complications of Chikungunya infection in patients from La Reunion island during the same period [50].

In 2006, all the military policemen deployed in Reunion Island during the chikungunya outbreak were enquired, 25% self-declared chikungunya infection (CHIK+) and 19% had positive serology. In 2008, a self-questionnaire was sent to the same persons, 403 responded, 101 were CHIK+. The latter presented higher frequency of rheumatic disorders and significantly lower quality of life (QoL) than non-infected (CHIK-) responders, 30 months after the outbreak. Six years later, the 646 participants to the 2006 enquiry were sent self-questionnaires by postmail with informed consent to the present study and proposal of new biological testing. Only 609 could be reached, and 252 fulfilled the questionnaire: 81 CHIK + (32%) and 171 CHIK-. The results are based on their declaration. CHIK + patients declared higher health care consumption between 2008 and 2012. They complained of more frequent and intense joint pain (40% versus 22% at least once a week, 64% versus 38% moderate to intense pain), stiffness and swelling, more frequent fatigue, headache and depress mood (respectively 60% versus 32%, 42% vs29% and 21vs 6%, p < 0.001). All dimensions of QoL were significantly impaired in CHIK + patients, reflecting social, physical and mental impact of the disease. The large differences observed in rheumatic morbidity, fatigue, and QoL lead to the conclusion that CHIK infection has very long term impact on health, social life and QoL with a very low proportion of patients returning to their previous health status.

Following the acute febrile polyarthritis, miscellaneous long-lasting rheumatic musculoskeletal disorders (RMSKD) are reported, consistent with chronic inflammatory rheumatisms (CIR) notably rheumatoid arthritis (RA). The post CHIK-infection stage remains a challenge to treat, while the early use of disease-modifying antirheumatic drugs (DMARDs) is recommended in RA. After the 2005-2006 CHIK outbreak in Reunion Island, CHIK-related RSMKD were retrospectively categorized and the empirical use of DMARDs starting with methotrexate (MTX) to treat the CIR forms was analyzed. The medical files of patients referred to rheumatologists in Saint Denis between 12/2005-05/2012 for persisting rheumatic disorders (>4 months) after a proven CHIK infection were reviewed [50]. De novo post-CHIK RMSKD was distinguished from exacerbation of pre-existing disorders. Among 159 patients included, 122 suffered from de novo RSMKD. RA accounted for 40/92, spondylarthropathy for 33/94, and distal undifferentiated polyarthritis for 21/94. Seventy-two patients were treated with MTX, reaching efficacy in 54 cases, versus failure in 18 cases. No severe side event was observed. The group “efficacy” was significantly associated with the early introduction of MTX within the first year of CIR progression (p =0.034).

## Conclusion

This meeting was a strong reminder of the efforts made in different fields such as drug development with more potent and better tolerated compounds and also new types of delivery system which may lead to both preventative and therapeutic use. The effort to end the epidemic is now including multipronged interventions while the eradication paradigm remains well and alive with in-depth viro-immunological studies and new pharmacological and gene therapy interventions. On the other hand, HCV infection is the first chronic viral disease that we are now able to cure but the same issues for “test and treat” as for HIV remain.

## Competing interests

The authors declare that they have no competing interests.

## Authors’ contributions

All the authors participated equally to the article. All the authors read and approved the final manuscript.

## Special note

We dedicate this paper to Professor Joep Lange who died on July 17 in the crash of flight MH17.
